# Middlesex Hospital

**Published:** 1830-09

**Authors:** 


### HOSPITAL REPORTS.

### MIDDLESEX HOSPITAL.

### *Obliterated Urethra, with Operation.*

————, æt. thirty, was admitted into the Middlesex Hospital on the 16th of July, at seven in the evening, labouring under retention of urine. His business had been that of porter to a coachmaker: his work he thought had been too hard for him; but it was not till a fortnight before his admission that he could recollect with certainty having experienced difficulty in voiding urine. At that time a change took place in his manner of living: his circumstances happened to have improved, and, instead of starving upon insufficient nutriment, he now had it in his power to live well, and he lived freely and intemperately. Symptoms of obstruction of the urethra became apparent on this change of diet: he passed urine not without great effort and straining, and in a small stream, or by drops; his sufferings became aggravated, and for the twenty hours preceding his admission, no water had come from him.

Some attempts had been made during that morning, for the first time, to pass an instrument into the bladder, but they were ineffectual; and it appeared, from the blood which had flowed, that the membrane of the urethra had been torn. Mr. Mayo, under whose care the patient fell, now tried to pass an instrument into the bladder, but without success: the catheter appeared firmly opposed by a stricture situated near the triangular ligament of the urethra, and, when pressed with more force, seemed to find a channel in the substance of the corpus spongiosum. The patient complained of pain in the hypogastrium; the belly, particularly at the lower part, was tumid, as if from a distended bladder, but, when it was examined by the rectum, the bladder did not feel large or full. The pulse was under 100; the tongue furred, but moist; the countenance anxious.

The patient had forty drops of laudanum administered to him, and was placed in the warm bath: he experienced considerable relief, and was persuaded that he had passed water while he was in the bath.

At eleven o'clock P.M. Mr. Mayo saw this patient again: he appeared composed, and had lost the restlessness and anxiety which had before existed. The bladder was not tumid. Sixteen leeches was ordered to be applied to the perineum, and an injection administered containing a drachm of laudanum.

At four in the morning, the patient, who had dozed occasionally during the night, became again distressed, and his breathing hurried. Mr. Mayo was sent for, and, observing no symptoms that were not referrible to the urinary organs, and being unable to pass an instrument along the urethra into the bladder, with the consent of his colleagues proceeded without delay to divide the obstruction. The operation was performed in the following manner: A common staff was passed into the urethra, as far as it would go, to near the ligament of Camper; an incision, an inch in length, was then made parallel to the raphe of the perineum, and the groove of the staff cut into, close upon the obstruction; the staff was then withdrawn a little, and the scalpel carried forward half an inch in the direction of the urethra. The staff was then withdrawn, and a middling-sized silver catheter was passed with facility along the urethra into the bladder. The operation occupied about a minute. The quantity of water that flowed through the catheter amounted to no more than six ounces: it was slightly turbid, or contained a little mucus. The patient was placed in bed again; but he did not appear relieved by the operation: he had hiccup, his breathing became more hurried, he sank, and died at twelve o'clock.

The following day the body was carefully examined. The brain, the heart, and lungs, and the abdominal viscera and the kidneys, were perfectly free from disease. The only morbid appearances were in the bladder and urethra. The bladder was something thicker than usual, and towards the fundus, and in front, there was a round hole, that looked like a phagedenic ulcer: this aperture was much larger upon the outer than upon the inner surface of the bladder; the outer diameter being three fourths of an inch, that towards the mucous surface of the bladder only one third of an inch.

The cellular membrane between the peritoneum and linea alba, in front of this aperture and over an extent of sixteen inches square, was loaded with dark serous exudation, but which had not a urinous smell.

The state of the urethra was remarkable: it appeared evident that the incision into the groove of the staff had exposed that instrument, *not in the urethra*, but in the spongy body, where, for three fourths of an inch, it lay in a false passage. The next and last incision had opened this false passage into the membranous part of the urethra beyond, thus establishing a temporary free and continuous channel from the orifice of the urethra to the bladder; which channel, had the patient lived, would no doubt have closed towards the perineum, and the urethra have been restored. What completed the interest of this examination was the following circumstance: the final obstruction had consisted, *not in a narrow*

***stricture,*** but in ***an absolute obliteration*** of the canal of the urethra, for the length of half an inch. The staff having made its way into the substance of the spongy body beyond the stricture, when it was cut into, the closed part of the urethra was left untouched behind the incision, allowing the facts that have been mentioned to be distinctly verified.

The parts are preserved in Mr. Mayo's museum. There is no thickening about the obliterated urethra, which, from the state of a narrow stricture, must have passed into absolute obstruction in less than twenty-four hours.

---

### *Strangulated Crural Hernia. Operation.*

Eliz. Gibbs, æt. 52, was admitted into the Middlesex Hospital, in the evening of the 16th of July, under the care of Mr. Mayo. For sixteen years she had been subject to rupture in the left groin, and during the greater part of that time she had worn a truss. The 14th of July had been a day of hard work with her, in her occupation of washing; at eleven at night she was obliged to desist from work, as she turned sick, and observed that the rupture had come down, and was swollen under the truss: various attempts were made ineffectually to reduce it, but it was not till the rupture had been two days in this state that she came to the hospital. An injection was now administered: she was placed in the warm bath, and continued attempts were again made to return the bowel, but fruitlessly. The tumor was not very tense; but the pressure employed gave pain, and there was tenderness of the belly, immediately above the left groin. The patient occasionally vomited.

Under these circumstances, Mr. Mayo recommended the patient to submit at once to the operation. Upon her refusal, now late at night, he left her, desiring that another injection should be administered.

The following morning the symptoms had become more urgent: the vomiting was more frequent, the countenance appeared distressed, and the belly was more painful on pressure. A tobacco injection was now administered, and, although it produced no effect upon the hernia, yet the faintness which it occasioned for the first time alarmed the patient for her safety, and she consented to the operation.

The operation was performed about two o'clock on the 17th. The contents of the sac was a fold of small intestine, of a dark colour, but neither sphacelated nor its surface inflamed: it was returned. A partial tenderness of the belly remained for five days, for which leeches and fomentations were applied three times, with benefit. The bowels were freely opened with castor oil the day after the operation. The incision in the groin healed rapidly.

---

A few days ago we saw Mr. Mayo perform the operation of lithotrity upon a patient in the Middlesex Hospital: the case is going on very satisfactorily. We do not enter into the details, as Mr. Mayo has promised us an original communication upon this subject, containing an account of this case with others. As the success of lithotrity depends not only upon the expertness with which the operation is performed, but also upon the previous and subsequent treatment of the patient, we are glad to see it taken up with zeal by those whose general surgical talent gives to their manual dexterity the best possible chances of success.

